# Recording Risk Factors of Physical Abuse in Children Younger Than 36 Months With Bone Fractures: A 12-Years Retrospective Study in an Italian General Hospital Emergency Room

**DOI:** 10.3389/fped.2020.00183

**Published:** 2020-04-21

**Authors:** Miriam Castagnino, Alessandra Paglino, Carla Berardi, Sara Riccioni, Susanna Esposito

**Affiliations:** ^1^Department of Surgical and Biomedical Sciences, Pediatric Clinic, Università degli Studi di Perugia, Perugia, Italy; ^2^Primary Care Pediatrician, Perugia, Italy; ^3^Radiology Unit, Santa Maria della Misericordia Hospital, Perugia, Italy; ^4^Department of Medicine and Surgery, Pediatric Clinic, Pietro Barilla Children's Hospital, University of Parma, Parma, Italy

**Keywords:** child abuse, emergency room, bone fracture, skeletal fractures, skull fracture

## Abstract

Skeletal fractures (SFs) are very common in pediatrics. In some cases, they are secondary to child abuse. Differentiation of accidental from non-accidental fractures (NAFs) is essential as in abused children risk of further injuries leading to severe clinical problems and death is significant. Main objectives of this study were to evaluate the characteristics of SFs of children ≤3 years of age presenting to the Emergency Room (ER) of a Children's Teaching Hospital over a 12-year period and the attention paid by ER physicians to the identification of the indicators that increase suspicion of NAF and that suggest referring of the patient to the child protection agencies. This is a descriptive, retrospective study of the medical records of all the pediatric patients ≤ 36 months of age admitted to the ER of the Azienda Ospedaliera Santa Maria della Misericordia, University of Perugia, Perugia, Italy, for radiological documented SFs between January 1, 2004, and March 31, 2016. Available information was used to evaluate whether indicators of possible child abuse were documented by the ER staff and whether diagnosis of potential abuse was followed by further screening or referral to child protection agencies. During the study period, 11,136 accesses of the ER by children younger than 36 months were documented, among whom 417 presented long bone or skull fractures. Skull fractures were significantly more common among children <12 months of age (*p* = 0.001), whereas radius/ulna and humerus fractures were diagnosed significantly more frequently in children 12–36 months of age (*p* = 0.036 and *p* = 0.022, respectively). Recorded medical history was considered inadequate in 255 (61.2%) cases with no difference related to patient's age. Our study showed that the majority of charts in case of SFs were found to contain inadequate documentation to explain causes at the heart of the fractures and, therefore, to rule out any inflicted trauma. The development of specific referral guidelines, along with the continuous education and training of health professionals, as well as the preparation of structured medical forms, are essential measures to activate in order to improve the referral of children from the ER to child protection agencies.

## Introduction

Skeletal fractures (SFs) are very common in pediatrics. Results of most of the epidemiological studies in this regard have reported that SFs occur in about 20 children/1,000/per year, accounting for 10–25% of childhood injuries (1–5). It has been calculated that about one third of children is expected to have a fracture before 16 years of age (6). However, both incidence and type of fracture vary significantly according to age. They are more common during school age and adolescence although they can be diagnosed in younger subjects with an incidence of ~0.5% among infants and 1.7% among children aged 1–2 years (7). Moreover, clavicle and distal humerus are the bones more commonly injured in younger children, but with increasing age fractures of the distal radius and hand are more commonly seen (6).

Accidental traumas are the most common cause of SFs in children. However, in some cases, they are secondary to child abuse. Differentiation of accidental from non-accidental fractures (NAFs) is essential as in abused children risk of further injuries leading to severe clinical problems and death is significant. It has been shown that if diagnosis of abuse is not identified and no intervention is carried out injuries can recur in about one third of the cases with death in 5–10% of them (8). However, distinction is not easy, particularly in younger children who are too young or afraid to report the violence suffered. Moreover, frequently guilty parents, or caregivers tend to distract the doctor from the idea of violence by offering false but rationally acceptable explanations for the SFs. Finally, abuse can be missed because questions regarding description of the injury and social concerns that favor abuse are not asked by physicians. It was shown that at least 20% of abusive fractures is not identified with values even higher when only younger children are considered. This explains why a number of practical guidelines to differentiate abusive from accidental SFs have been prepared (9, 10). However, patient assessment for abusive SFs is often suboptimal (11–13). Main objectives of this study were to evaluate the characteristics of SFs of children ≤3 years of age presenting to the Emergency Room (ER) of a Children's Teaching Hospital over a 12-year period and the attention paid by ER physicians to the identification of the indicators that increase suspicion of NAF and that suggest referring of the patient to the child protection agencies.

## Materials and Methods

This is a descriptive, retrospective study of the medical records of all the pediatric patients ≤ 36 months of age admitted to the ER of the Azienda Ospedaliera Santa Maria della Misericordia, University of Perugia, Perugia, Italy, for radiological documented SFs between January 1, 2004, and March 31, 2016. This hospital is an urban tertiary teaching institution with more than 10,000 pediatric ER admissions every year.

Patients were identified by searching for ICD-10 codes. Charts were reviewed by two of the authors (MC and AP) and radiological images were all analyzed by an expert radiologist (SR) blinded to medical information. All the data reported in the charts concerning triage, ER ledgers, nurse and physician notes, and social work notes were collected. Demographic characteristics of each patient such as age and gender, presenting complaint, mechanism and time of injury, other historical and examination data, and fracture characteristics (i.e., name of the fractured bone, type, and location of the fracture) were taken into account. The skull fractures were classified as linear, diastatic, and depressed. Location of long bone fractures was classified as physeal, metaphyseal, or diaphyseal (14).

Available information was used to evaluate whether indicators of possible child abuse were documented by the ER staff and whether diagnosis of potential abuse was followed by further screening or referral to child protection agencies. Attention was focused to the nine indicators that have been previously considered very effective in distinguishing inflicted fractures from NAFs (15–24). These included incompatible or inconsistent history (value = 1), unreasonable or unexplained delay in presentation (value = 2), all unwitnessed injuries (value = 3), children under 1 year of age with a fracture (value = 4), all patients with other injuries suspicious of abuse and a long bone fracture (value = 5), previous presentation to the ER with a fracture (value = 6), previous presentation to the ER with more than one injury during the study (value = 7), all patients with more than one fracture during the study period (value = 8), and high-risk fractures (ribs, vertebral, non-supracondylar fracture of the humerus under 18 months, femur fractures in children under 1 year of age, complicated skull fractures, metaphyseal “bucket-handle” and “corner fractures,” old or healing fractures; value = 9).

All the collected data were divided according to age patients in two groups, those of children ≤ 12 months and those of children aged >12 months. All the data were analyzed with Student's test and the χ^2^ test using STATA14 software.

## Results

Between 2004 and 2016, 11,136 accesses of the ER by children younger than 36 months were documented: 411 patients out of 417 accesses (98.6%) presented long bone or skull fractures during the study period (6 patients were evaluated twice). The mean patient age ± standard deviation was 22.8 months ± 8.5, and the age range was between 1 and 36 months. Among these, 205 patients (49.2%) were female, 54 patients were younger than 12 months (12.4%), and 223 out of 417 children (53.5%) were hospitalized. No significant differences in the number of SFs per year were observed over this time.

In Table 1 the type of SFs diagnosed in the two study groups is listed. In 157 (37.6%) cases, they were of the radius/ulna, 82 (22.3%) clavicular, 55 (13.2%) of the tibia/fibula, 42 (10.1%) of the skull, 40 (9.6%) were humeral and 33 (7.9%) femoral. In 8 (1.9%) cases multiple fractures (2 skull + clavicle, 1 radius + clavicle, 1 humerus + ulna, 1 humerus + radius + ulna, one heel + tibia + fibula, 1 skull + clavicle + first rib) were evidenced. Skull fractures were significantly more common among children <12 months of age (*p* = 0.001). On the contrary, both radius/ulna and humerus fractures were diagnosed significantly more frequently in children 12–36 months of age (*p* = 0.036 and *p* = 0.022, respectively).

**Table 1:** Type of skeletal fractures (SFs) diagnosed in the study population according to patient's age.

**Type of SF**	**Age <12 months (*n* = 54)**	**Age 12–36 months (*n* = 363)**
Radius/ulna	7 (12.9)^∧^	150 (41.3)
Humerus	1 (1.9)^∧^	39 (10.7)
Clavicle	10 (18.5)	72 (19.8)
Tibia/fibula	5 (9.3)	50 (13.8)
Femur	4 (7.4)	29 (8.0)
Skull	25 (46.3)^*^	17 (4.7)
Skull complicated	2 (3.7)	6 (1.6)
Multiple complicated	2 (3.7)	6 (1.6)

*Numbers (percentages in parenthesis)*.

∧ p < 0.05 and

* *p = 0.001 vs. age 12–36 months; no other significant differences between the groups*.

Table 2 shows the type of history reported by the parents for the occurrence of SFs. at ER admission according to patient's age. Free-time and domestic injuries were the most common reasons for SFs in our study population, with no difference related to age.

**Table 2:** Type of history reported by the parents for the occurrence of skeletal fractures (SFs) at Emergency Room (ER) admission according to patient's age.

**Type**	**Age 12–36 months (*n* = 363)**	**Age <12 months (*n* = 54)**
Domestic injury	18 (33.3)	133 (36.6)
Free time injury	23 (42.6)	157 (43.3)
School injury	2 (3.7)	12 (3.3)
Motor-vehicle crash	1 (1.9)	3 (0.8)
Other	2 (3.7)	16 (4.4)
Not recorded	8 (14.8)	42 (11.6)

*Numbers (percentages in parenthesis)*.

*No significant differences between the groups*.

Although in the majority of the cases the type of history was reported, the exact injury mechanism, such as falls from stairs, beds or other furniture, fall during well-defined play or from parents' arms, was documented overall only in 76 (18.2%) out of 417 patients. Recording of whether the injury was witnessed occurred in 41 (9.8%) children and in 7 (17%) it was assessed as unwitnessed. The time elapsed between the SF occurrence and presentation to the ER was recorded in 120 (28.8%) children and an unexplained delay was indicated in 7% of the cases. Considering together all these findings, recorded medical history was considered inadequate in 255 (61.2%) cases with no difference related to patient's age.

Table 3 shows indicators of child abuse registered at ER admission according to patient's age.

**Table 3:** Number of indicators of child abuse registered at ER admission according to patient's age.

**Risk indicators for abuse (*n*)**	**Age <12 months (*n* = 54)**	**Age 12–36 months (*n* = 363)**
0	32 (59.2)^*^	318 (87.6)
1	19 (35.2)^*^	29 (22.8)
2	3 (5.6)	12 (3.2)
3	0 (0.0)	2 (0.6)
4	0 (0.0)	2 (0.6)

*Numbers (percentages in parenthesis)*.

* *p < 0.05 vs. age 12–36 months*.

*No other significant differences between the groups*.

In 67 out of 417 (16.1%) accesses to the ER, at least one risk indicator was observed. In 15 out of 417 (3.6%), 2 out of 417 (0.5%) and 2 out of 417 (0.5%) accesses, two, three and four indicators were identified, respectively. When analyzing the subgroup of children <12 months of age, 18 out of 54 (33.3%) patients were found to present one risk indicator, whereas 3 out of 54 (5.6%) patients presented more than one risk indicator (*p* < 0.05 vs. age 12–36 months).

None of the children were referred to child protection agencies for further evaluations.

## Discussion

Our study showed that ER documentation of pediatric injury lesions is quite insufficient, making child physical abuse very difficult to suspect. In our analysis, we noted: (1) the absence of a documented clinical history to raise the suspicion of physical abuse (presence/absence of witnesses, mechanism of injury, etc.); (2) the presence of a general standardized causal code in almost all medical records examined; (3) the absence of a structured medical form to use as a checklist that might be helpful in the identification of abuse; (4) the poor radiological documentation in the identification of specific elements of abuse (such as metaphyseal lesions, corner fractures, etc.); (5) the compelling need for persistent education of health care professionals (25–29).

The types of fractures at high risk for being related to child physical abuse vary from study to study. Several studies compared fracture types among young children to distinguish accidental fractures from non-accidental ones (27–29). The authors of these studies agreed that humeral fractures are most likely caused by physical abuse, especially in children younger than 12 months. Similarly, other authors claimed that a femoral fracture in a non-ambulant child is suspicious (30, 31). After comparing patients younger and older than 12 months, we observed a statistically significant difference in: (1) fracture sites, i.e., the skull was the most common site of fracture in children <12 months of age, whereas the humerus was the most common site of fracture in children over 12 months of age; (2) the presence of a family history, i.e., ER operators documented the history of the youngest group of patients better than that of the oldest group; (3) the presence of witnesses, i.e., this information was mostly reported in children younger than 12 months of age; (4) the delay in access to the ER.

In our study, the group of children younger than 12 months accounted for 45.5% of all high-risk fractures. Our data, however, do not allow a feasible estimation of the probability that the children included in the studies are actual victims of abuse. Nevertheless, considering only fractures thought to be highly specific for child abuse, the suspicion of inflicted trauma might not be ruled out, but indeed, it should be either excluded or confirmed. Furthermore, in the subgroup of children <12 months of age who are at the highest risk of abuse, more than 30% of patients were found to have at least one risk indicator besides age and to be subjected to further diagnostic investigations.

Our retrospective study analyzed a high number of cases over a long period of time. However, the retrospective nature of the present study has limited our findings because it was not possible to know if the suspected cases were real abuse cases or not. We may only assess the history and examination based on the documentation available in the medical records. We were not able to ascertain how many cases of child abuse did occur in the sample population as well as we did not develop a structured medical form to be used as a checklist for child abuse identification. In addition, data have been collected in a single hospital and should be confirmed in different hospitals and different countries.

## Conclusions

Our study showed that the majority of charts in case of SFs were found to contain inadequate documentation to explain causes at the heart of the fractures and, therefore, to rule out any inflicted trauma. Doctors in the ER may miss the indicators of abuse when the clinical history and medical examinations are not comprehensively evaluated. Therefore, the knowledge of indicators raising the suspicion of abuse should be implemented. The development of specific referral guidelines, along with the continuous education and training of health professionals, as well as the preparation of structured medical forms, are essential measures to activate in order to improve the referral of children from the ER to child protection agencies.

## Data Availability Statement

All datasets generated for this study are included in the article/supplementary material.

## Ethics Statement

The studies involving human participants were reviewed and approved by Ethics Committee of Umbria Region (PED-2019-001). Written informed consent to participate in this study was provided by the participants' legal guardian/next of kin.

## Author Contributions

MC and AP co-wrote the first draft of the manuscript and participated in patient's management. CB gave a scientific contribution. SR performed the radiological evaluation. SE critically revised the paper and gave her scientific contribution. All the authors read and approved the final version of the manuscript.

## Conflict of Interest

The authors declare that the research was conducted in the absence of any commercial or financial relationships that could be construed as a potential conflict of interest.
